# Robust and Democratic s‑SNOM Data Analysis and Modeling in Quasar

**DOI:** 10.1021/acsomega.6c02534

**Published:** 2026-05-27

**Authors:** Gergely Németh, Marko Toplak, Stuart Read, Raul de Oliveira Freitas, Ferenc Borondics

**Affiliations:** † 482623 SOLEIL Synchrotron, L’Orme des Merisiers, RD128, Saint Aubin 91190, France; ‡ 172648 Wigner Research Centre for Physics, 29-33 Konkoly-Thege str. Budapest 1121, Hungary; § Faculty of Computer and Information Science, 117197 University of Ljubljana, Večna pot 113, SI-1000 Ljubljana, Slovenia; ∥ 42476 Canadian Light Source, Inc., 44 Innovation Boulevard, Saskatoon, Saskatchewan S7N 2 V3, Canada; ⊥ Brazilian Synchrotron Light Laboratory, 55536 Brazilian Center for Research in Energy and Materials, Campinas, SP 13083-970, Brazil

## Abstract

Near-field optics is a blooming field of physics with multiple techniques that contribute to help understand novel and complex optical phenomena. Among them, scattering-type scanning near-field optical microscopy (s-SNOM) is a well-established method, which was largely enabled by the advent of high-brightness tunable or broadband infrared sources. Thus, today, besides imaging, spectroscopy, and hyperspectral measurements are easily available at the nanoscale. Such instrumental capabilities and the commercialization of s-SNOM instruments made experiments on low-energy phenomena accessible to a large number of laboratories. However, an important outstanding issue is the lack of data analysis tools. Underlying phenomena are complex and require flexible, experiment-specific workflows, numerical modeling, or the use of artificial intelligence. While expert research groups develop specific, in-house processing codes, adapting, exchanging, and reproducing data analysis workflows even within those laboratories can be challenging. Therefore, the need for software that allows easy data processing and interpretation is clear. Here, we introduce the first open-source software suite based on visual programming that democratizes s-SNOM data analysis, unlocks the potential of machine learning for newcomers to the field, and empowers experts to develop and collaborate more efficiently, benefiting the whole near-field community.

## Graphical abstract

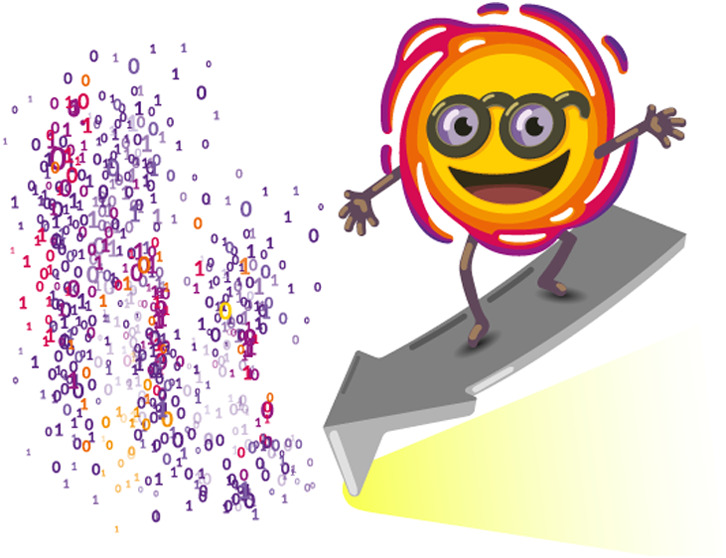

## 1 Introduction

Infrared (IR) spectroscopy and spectromicroscopy are essential tools for material characterization, understanding optical properties, structure determination, and various phenomena across biology, chemistry and physics. The technique holds a long and rich history, therefore vast commercial and open-source databases and data analysis software exist. Scattering-type scanning near-field optical microscopy (s-SNOM) is a newer technique compared to far-field infrared spectroscopy that capitalizes on the ability of atomic force microscopes (AFMs) to probe material-light interaction in the near-field. IR s-SNOM instruments are commercialized today by several companies but none of them provide a comprehensive, flexible, and open-source data analysis tool. Experimentally, IR s-SNOM is an established technique widely used across many subfields of physics, chemistry, and biology.^1–3^ Many advanced use cases of IR s-SNOM are examples in low-dimensional material research and nanophotonics, often using custom-built setups including cryogenic temperature and/or magnetic field.^4–6^ With the commercialization of IR s-SNOM imaging and spectroscopy (a.k.a. nano-FTIR), the method also found applications in nanoscale analytical research. For example, in polymer science to identify nanoplastics,^7–9^ in material science to explore the effect of nanocorrosion,^10^ or nanoscale changes in oxide layers.^11,12^ Recently, the possibility to retrieve nanoscale label-free chemical information, elevated IR s-SNOM into the forefront of biological research. It enabled studies of single protein strands, lipid bilayers, biominerals, and even living cells.^13–15^ This broad scientific landscape shows that the IR s-SNOM technique is extremely versatile. With this wide scope, it is inefficient for various users to repeatedly reimplement or reconsider the fundamental aspects of data processing. This applies to both expert groups from nanophotonics and especially users aiming for nanoanalytics, where s-SNOM is but one tool among the multiple techniques in a complex study. Still, there are no freely available, easy-to-use, and versatile tools for data analysis.

Below, we introduce a software package based on the principles of visual programming,^16^ a paradigm that has existed for multiple decades in programming and instrument control, for solving the complex task of s-SNOM data analysis. Visual programming enables the rapid development of problem-specific workflows for custom instruments or data analysis in scientific laboratories or industrial settings. Thus, users avoid complex coding tasks and replace them with intuitive, visual interactions to build workflows by connecting components representing data, calculations, or visualizations. The visual programming approach facilitates data processing and makes the implementation and comprehension of complex workflows more accessible to scientists without the need for a programming background. These ideas found their way to data analysis and education, where the flexibility of workflows is paramount and the focus of the problem is at a higher level than writing computer code. Therefore, providing a reusable, configurable component on a canvas enables the implementation and sharing of quick and efficient data analysis.

Modern s-SNOM instruments can operate in imaging (fast spatial scanning) or spectroscopy (fast collection along an energy axis) modes and consequently acquire single images, image stacks, or various types of spectroscopy data sets (see Figure 1). Today, there is no software that can handle all these data types and, at the same time, provide specific correction methods in an open-source, user-friendly package. For s-SNOM imaging, researchers default to using scanning probe microscopy software like Gwyddion,^17^ which, although having powerful general functionality, might be incapable of processing complex-valued data, lack s-SNOM -specific functions, or batch processing. Such limitations often prevent complex studies, such as performing proper multispectral laser imaging data analysis, or the data processing slows down to the manual processing level. With the automation of both measurement and data processing, valuable insight could be developed to complex data, but these are missing from studies even today. Some limitations can be addressed by creating custom code and processing workflows, but UI-dependent functionalities are cumbersome to implement and quickly become unsustainable for elaborate processing workflows.

**Figure 1:**
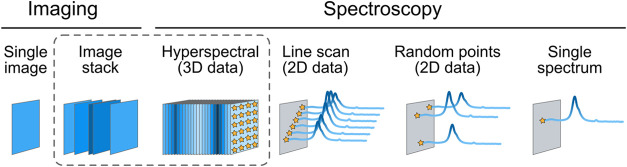
Data collection schemes in modern s-SNOM instruments. Color brightness represents signal intensity, stars mark measurement positions. For clarity, we omitted spectra on the 3D hyperspectral data set.

A different bottleneck appears for analyzing broadband spectroscopy data sets from nano-FTIR measurements. Currently available codes are missing simple functionality such as visual correlation of imaging data and spectra, or slicing and processing of hyperspectral data sets, and performing spectrum and image processing at the same time.

To solve these problems, we released the *orange-snom* add-on for Quasar, an open-source data analysis software that extends the Orange data mining platform^18,19^ with spectroscopy-specific data processing tools. *Orange-spectroscopy*,^20^ the main add-on of Quasar, contains a variety of visual components (widgets) implementing the most important data analysis tools for spectroscopy and spectromicroscopy. Building on Orange, it brings together spectroscopy data with multivariate data analysis and machine learning. With the new extension, we provide s-SNOM and nano-FTIR-specific widgets and functionality to enable end-to-end data processing in the same environment for nanospectroscopy data using visual workflows, and bring machine learning to the nanoscale with the ease of a few clicks instead of tedious coding. Importantly, Quasar also helps avoid unintentional mistakes in the implementation of workflows, as the components are built in a way that provides a structure and methodology for correct, scientifically and logically consistent data analysis. Below, we will provide several case studies that solve some of the most common data analysis problems for s-SNOM data sets, which can also help expert users to get started with building more complicated workflows.

## 2 Results and Discussion

In this section, we showcase examples covering data loading, manipulation, and plotting as well as complex processing. Since the functionalities mainly rely on the methods implemented on a stand-alone *pySNOM* package, the GUI-independent part of processing can be done with Python scripting.^21^ To demonstrate this, Jupyter notebooks and the data sets of all case studies, implementing the same functionality, are shared in a GitHub repository.^22^ Upon installation, a requirements file is followed to handle the correct package and dependence versions. For the workflows below, we used *pySNOM* 0.3.0 and *snompy* 0.1.9. In the development, we also made sure that the workflows remain compatible with newer releases.

Note that the aim of the case studies presented below is to introduce and discuss several s-SNOM specific workflows. If the reader would like to familiarize themselves with the working principles of Quasar or Orange in detail, we refer to previous publications,^18,20^ Web sites,^23^ and online tutorials.^24^

All data sets used in the following studies were measured with a commercial near-field microscope (IR neaSCOPE+, Attocube GmbH, Haar, Germany) in imaging s-SNOM or nano-FTIR mode. In the s-SNOM imaging mode, the microscope works in pseudoheterodyne detection mode^25^ using a tunable monochromatic infrared quantum cascade laser (MIRcat QCL, Daylight Solutions, San Diego, CA, USA). This source consists of four laser chips covering the 920–1740 cm^–1^ spectral range. In the case of broadband nano-FTIR measurements, we utilize the infrared light for a bending magnet device at the SMIS beamline of SOLEIL synchrotron. In both measurement modes, HgCdTe detectors are used (Infrared Associates and Kolmar technologies). Detailed image and spectrum acquisition parameters can be found in the corresponding sections.

### 2.1 Case Study 1—Basic Data Exploration

In an s-SNOM experiment, visual camera images, both optical and mechanical signals, and various spectroscopy data (see Figure 1) are collected at the same time. As the technique is strongly geared toward spatial representation of the sample through these various signals, it is essential to enable a visual correlative exploration of the data set. Below we show a basic example of loading and displaying a nano-FTIR measurement, including the map image and the spectra.

The basic building blocks of a Quasar workflow are widgets that implement a specific operation on the data. Widgets pass data to each other through channels that are represented by the connecting line between them. Whenever the data changes in one of the widgets via a parameter change, the new data flows through the entire workflow, which creates an easy, intuitive way of working.[Fn fn1]

A workflow is shown on the top-left part of Figure 2. It implements a solution for the correlative display and visual inspection of images and nano-FTIR spectra at specific points. Here, we use data containing multiple point spectra, white-light, and AFM images, which were used during the measurement to select locations. The ***File*** widget loads imaging data, while the corresponding spectroscopy data sets are loaded by ***Multifile***.[Fn fn2]

**Figure 2:**
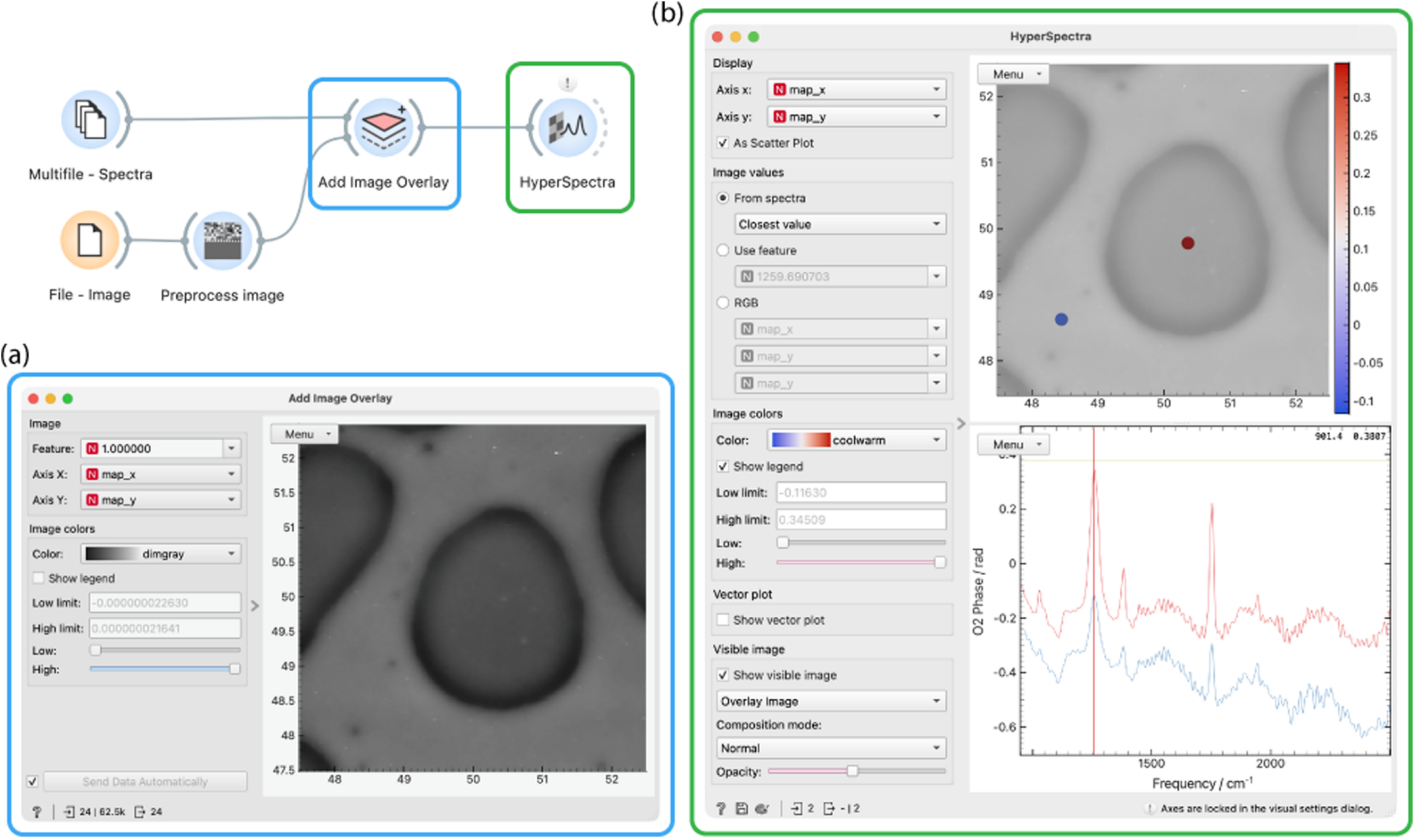
Top-left: workflow for simple data exploration and display. ***Multifile*** is used to load nano-FTIR spectra, while the ***File*** widget loads an AFM image that is passed to ***Preprocess image*** to correct the plane level. (a) The ***Add Image Overlay*** widget allows the customization of the overlay image, including color map and scaling, and combines the image with the spectroscopy data set. (b) In ***Hyperspectra***, the “*As Scatter Plot*” option depicts the locations of the spectra on top of the overlay image and allows the selection of single or multiple points.

Subsequently, they are combined into a single data set by the ***Add Image Overlay*** widget (Figure 2a). The user can customize the look of the overlay image by changing the colors or the display limits. The generated image is added to the “*Visible image*” attribute of the data table containing the spectra. Finally, we can display the combined data together in ***Hyperspectra*** (Figure 2b) with the image on top showing positions of single point spectra and the spectra themselves on the bottom panel. Enabling the “*Show visible image*” option displays the overlay data, and using the “*As Scatter Plot*” option for the image values allows one to showcase and interactively view spectra from different locations. Importantly, selections on either the top (image) or bottom (spectra) panel can be performed and propagated to further data analysis.

This workflow is also suitable for displaying slices or integrals of hyperspectral cubes on top of an AFM map, but ***Hyperspectra*** can be used alone for data exploration.

### 2.2 Case Study 2—Interferogram Processing

One of the challenges in nano-FTIR spectroscopy data processing comes from the asymmetric arrangement of the interferometer. In conventional FTIR interferometers, the setup consists of a fixed and a moving mirror, which provide the two beams for the interference, and the sample is located outside of the interferometer. However, in broadband IR s-SNOM instruments, the tip is located in the fixed mirror arm, resulting in an asymmetric arrangement.^26^ As a consequence, the measured interferogram shows a dispersive shape on the positive optical path difference side caused by the sample’s refractive index dispersion. These interferograms are heavily asymmetric and offset from the center point of the interferometer’s scanning range to maximize spectral resolution and decrease artifacts, and they require different processing from the conventional measurements, as detailed in the literature.^26^ In Quasar, we implemented the spectrum calculation for asymmetric complex interferograms, including asymmetric apodization and phase spectra corrections. We introduced the new functionality in the ***Interferogram to Spectrum*** widget of the *orange-spectrosopy* add-on.

As shown in Figure 2, after loading both sample and reference interferograms with the ***File*** widget, we use ***Select Rows*** to choose the “*O2*” channel for further processing.

The ***Interferogram to Spectrum*** widget automatically recognizes that the datatable contains both amplitude and phase spectra provided from the experimental files. As shown in Figure 3a, the “*ComplexFFT*” option is selected by default for this format. We have several apodization functions to choose from. All are compatible with asymmetric apodization. After the widget finds the zero path difference (ZPD) point of the interferogram using the chosen method from “*ZPD search*”, it applies the apodization both to the left (negative path difference) and the right (positive path difference) side of the interferogram. Since the ZPD point is not in the center, the apodization window becomes naturally asymmetric. In case of loading amplitude and phase separately, the user has to connect the amplitude data to the “*Interferogram*” and phase data to the “*Phases*” inputs.

**Figure 3:**
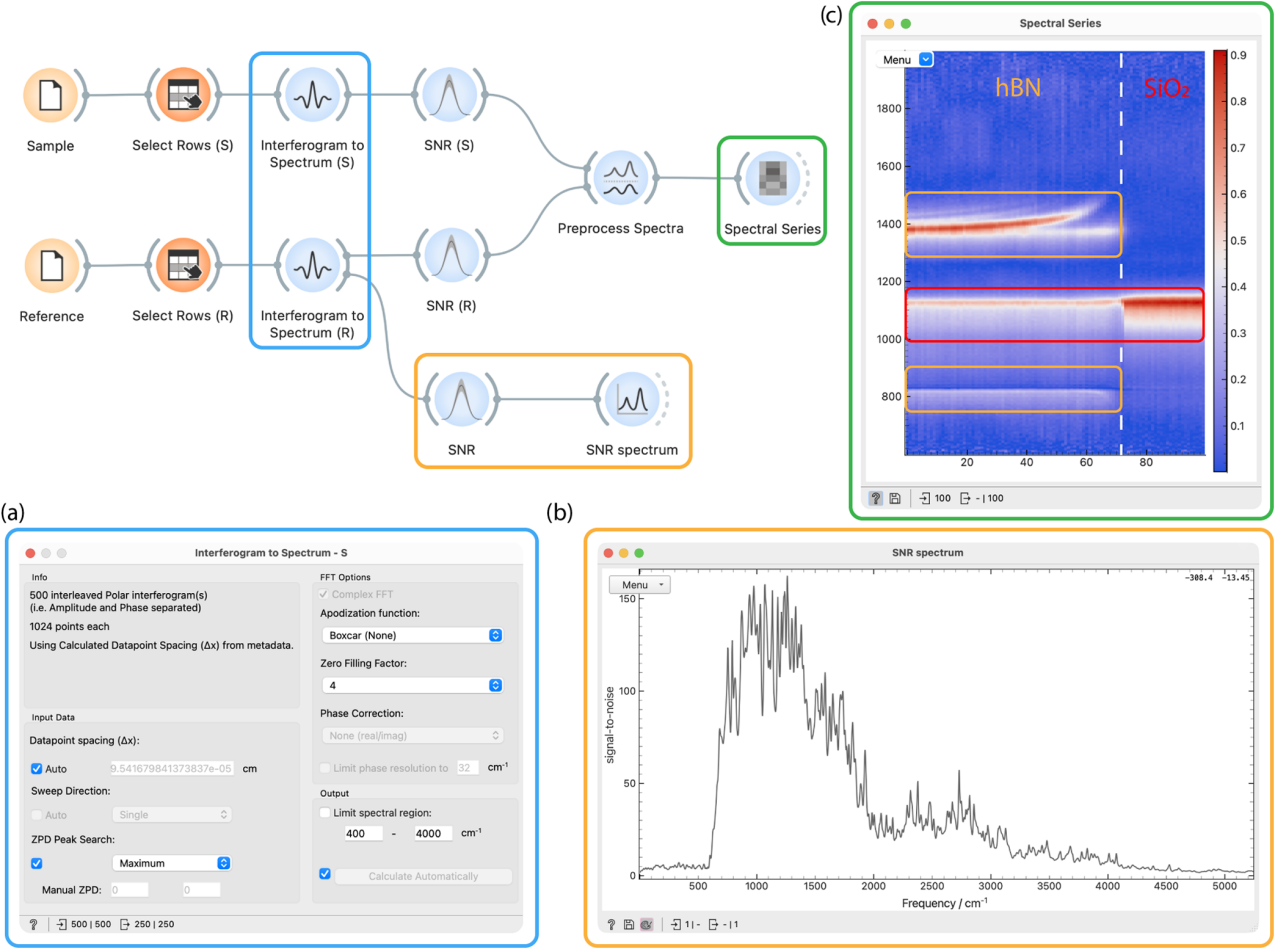
Top-left: workflow for interferogram processing. (a) The ***Interferogram to Spectrum*** widgets process the complex interferograms. (b) Spectral signal-to-noise ratio is computed by the ***SNR*** widget and plotted in a ***Spectra*** widget, here labeled as “*SNR spectrum*”. (c) The hyperspectral linescan normalized by the reference spectra is presented by the ***Spectral series*** widget, where the hyperbolic-phonon branches of the h-BN flake (orange rectangles) are clearly visualized.

Additionally, the ***Interferogram to Spectrum*** widget provides both the amplitude and phase spectra for the given data set. Both spectra are important in nanoFTIR spectroscopy, as amplitude represents local reflection and phase is related to the local absorption of the sample at the nanoscale.

To showcase the use of the ***Interferogram to Spectrum*** widget for hyperspectral data, we use interferogram data from a hyperspectral linescan measurement on a hexagonal boron nitride (hBN) flake exfoliated onto a SiO_2_ substrate. The line was set up perpendicular to the edge of the hBN flake. During the linescan, the tip approaches the edge of the flake, and it finishes on the SiO_2_ substrate while measuring a new interferogram every 20 nm. Measurements were done at the SMIS beamline of SOLEIL synchrotron, using synchrotron IR radiation as the light source. The spectral line scan consists of 100 points. At each point, we recorded 5 spectra with the acquisition parameters of 90 nm tapping amplitude, 490 μm scan distance. We sample the interferogram at 1024 points with 20.1 ms integration time per point.

The widget requires both amplitude and phase interferograms, which are readily available in the recorded files. Choosing the “*ComplexFFT*” option, the calculation uses the entire complex-valued interferogram for Fourier transformation. The user also has the usual option to choose from different apodization functions and define zero filling. The apodization function is positioned regarding the option in the ZPD search section. By default, the widget uses the step size in the data to calculate the frequency axis, but the user can override it manually.

Another advantage of our custom processing is that commercial software from manufacturers typically outputs averaged spectra across multiple runs. However, the ***Interferogram to Spectrum*** widget provides all the individual spectra at each point. The spectra can then be averaged by coordinate with the help of the ***SNR*** widget, but, as the name suggests, it can also be used to calculate spectral signal-to-noise ratio, providing important information about data quality, and to identify measurement points with poor stability. The spectral signal-to-noise for the reference measurement is presented in Figure 3b. Since the same widget can also be used to calculate the averages for each position, we use it to obtain the average spectra for each point along the linescan and the reference measurement. ***Preprocess Spectra*** widget is then used to apply the reference, add a linear baseline correction and cut the spectra for the desired spectral range. At the end, the ***Spectral Series*** widget displays the consecutive spectra as a 2D map, where the two axes are the spatial position along the line (horizontal) and frequency axis of the spectra (vertical).

In Figure 3c shows highly pronounced spectral signatures at 1360–1610 cm^–1^ and 760–825 cm^–1^ region belonging to upper and lower phonon Restrahlen bands of the hBN crystal, respectively. In the upper band, different branches of the hyperbolic phonon modes are clearly visible toward the edge of the flake (marked by the dashed line). The third spectral feature, in between the hBN phonon bands, is the contribution from the SiO_2_ substrate. When the tip reaches the off-flake points, the SiO_2_ peak is greatly enhanced, and the hBN signatures vanish. From the spacing of the hBN phonon branches, the wavelengths of the surface polariton can be measured to retrieve the dispersion relation of the slab polariton mode.^27,28^

### 2.3 Case Study 3—Multispectral Image Processing

Here, we present a complex workflow to showcase the capabilities of Quasar for advanced s-SNOM data processing. We use a set of single-wavelength s-SNOM images and assemble them into a multispectral data set for further processing.

The data set consists of 47 s-SNOM images of human blood-derived extracellular vesicles (EVs) drop-casted on a gold substrate.^29^ The images were taken in the 1505–1735 cm^–1^ spectral region with 5 cm^–1^ steps. We chose an area with a few individual vesicles. Each scan was 7.500 × 2.925 μm with 300 × 117 pixel resolution. The integration time per pixel was set to 7 ms, the tapping amplitude was 85 nm, and the laser power was maintained at 1 mW for all the wavelengths. Human samples were collected upon informed consent. The task here is to create a multispectral data set from single-wavelength images to analyze the spectra of individual vesicles.

After tuning to a new wavelength for a new s-SNOM image acquisition, the AFM stage suffers several microns of random drift. This results in the shift of the object in the area of measurement, requiring image realignment as part of the processing. As the first step, we aim to correct the drift by calculating the normalized cross-correlation between the consecutive images and cropping them to the size of the overlapping area using the ***Align Stack*** widget. As shown in Figure 4, the widget has two inputs: optionally (as here), the user can supply a data set of reference images. The widget is able to calculate the translations from a reference image set and apply the corrections to the images connected to the “*data*” input. Here, we used this option because the optical images have spectrally varying contrast, which can lead to failed or inaccurate image registration. The AFM mechanical amplitude images (M1A) are more suitable as a reference because they show the same contrast of the objects regardless of the wavelength of the s-SNOM measurement. Since they are measured simultaneously with the optical channels, they match pixel-perfectly. In Figure 4a, the ***Align Stack*** widget window shows both horizontal and vertical drift shifts for each image (frame).

**Figure 4:**
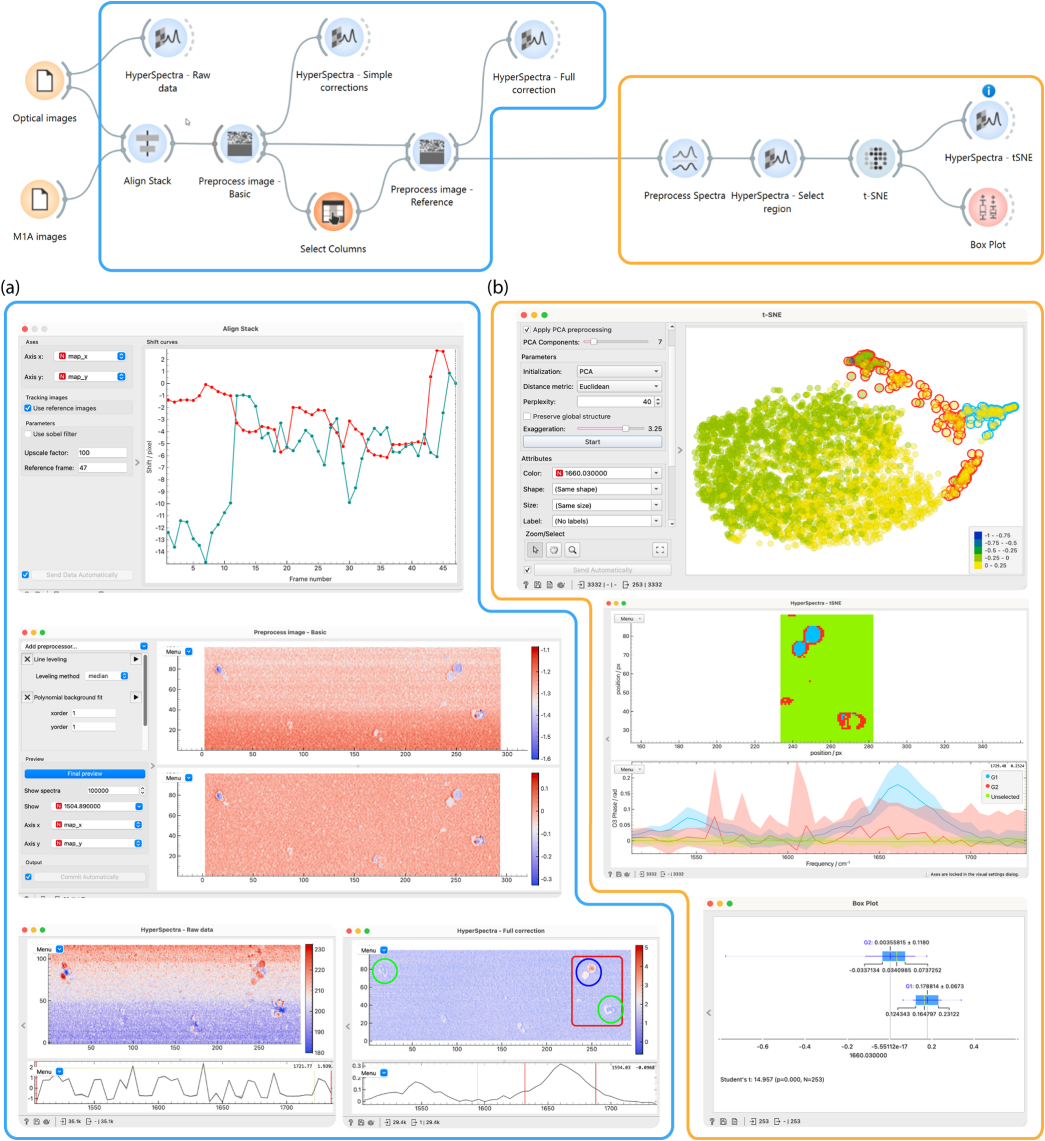
Example workflow for multispectral image processing (a) and subsequent machine learning analysis (b). In the blue frame, four widgets are highlighted: ***Align Stack*** corrects image drifts of measurements at different wavelengths and shows applied shifts in *X* and *Y* directions. ***Preprocess image*—*Basic*** removes measurement artifacts in the aligned stack using line leveling and plane subtraction. The ***Hyperspectra*** widgets are used to show the integrated spectral image of the data set and a spectrum from a vesicle before and after the drift correction. Subsequently, in part (b), the orange frame, the data is baseline corrected in ***Preprocess spectra***, cropped by ***Hyperspectra*** to the region marked by the red rectangle in (a), and analyzed with the ***t-SNE*** widget. A manual selection of two visually separated groups and their average spectra are plotted in ***Hyperspectra*—*tSNE. Box Plot*** shows statistical parameters at 1660 cm^–1^ for the two groups and runs a two-tailed *t* test to compare the distribution means.

At each wavenumber, the laser alignment was adjusted and the optical path optimized to maximize the optical signal. As a consequence, the raw pixel intensity values cannot be used to construct the spectrum; a reference is required to normalize the data. Thus, as a second step, we need to correct and normalize the images. In an ideal case, the substrate has a flat spectral response, which can be used for normalization. To obtain a meaningful spectrum, each image must be referenced to the substrate signal. Furthermore, any AFM instabilities and scanning artifacts have to be removed from each image. This usually involves line leveling and plane fitting, which are commonly used in AFM image processing. However, because the information from the measurements is complex-valued, amplitude and phase images have to be treated differently. Besides common scanning-probe image correction, s-SNOM -specific artifacts need to be corrected as well. The two most important corrections are on-pixel normalization and negative phase subtraction.^30,31^ Both are implemented in Quasar and accessible through the ***Preprocess Image*** widget, which works similarly to ***Preprocess spectra***. A sequence of corrections can be created that are applied in an ordered sequence from top to bottom of the list. Here, we applied line leveling and a polynomial background fit (**Preprocess image—Basic**). This way, all the values are normalized to the substrate level. In the second ***Preprocess image*** widget (**Preprocess image—Reference**), we selected a reference image at 1500 cm^–1^ using the ***Select Column*** widget to correct the negative phase contrast artifact often present in near-field images.^30^

In Figure 4, we show the full multispectral data analysis workflow where we combined the newly implemented s-SNOM specific corrections with the existing AI features from Orange. Part (a) of the figure highlights the most important steps of s-SNOM data preprocessing as well as its effect via integrated images of the raw and corrected data. The initial data shows a shifted pattern, resulting in uninterpretable spectra that appear like noise (Figure 4a—bottom left). The processed data set reveals the correct phase contrast of the vesicles and gives reliable multispectral information. As a consequence, one can clearly identify that some of the vesicles are protein-rich, and some are empty, even by simply looking at the image created from the integrated values of the Amide I peak at 1660 cm^–1^ (Figure 4a—bottom right). The ***Hyperspectra*—*Full correction*** shows the borders of integration to create the image. Low values in the image (blue) mean the integrated peak is missing from the spectrum; these vesicles (marked by green circles) do not contain proteins.

In Figure 4b, we go one step further in demonstrating the capability of the new tools combined with machine learning tools. The data set is cropped, baseline corrected and subsequently sent to a t-SNE analysis widget,^32^ an unsupervised algorithm to discover structure and reduce the size of multidimensional data sets. The result clearly shows at least two groups that separate from the main point cloud. After manual selection, they are shown in ***Hyperspectra*—*tSNE***. The large (unselected - green) group represents the substrate and the inside of some vesicle, as apparent from the group selection image in the ***HyperSpectra*—*tSNE*** widget. The corresponding vesicles are also indicated in (a) by the green circles. ***HyperSpectra*—*tSNE*** widget also features the average spectrum of all the spectra categorized in each group. The red point cloud selected (G2 group) in the ***tSNE*** widget corresponds to the edges of the vesicles. At the edges of small, well-defined objects, s-SNOM measurements often show artifacts, as evident in the averaged, very noisy spectra of this group. The most separated group (blue selection, G1 group) clearly shows the EVs that are rich in protein. (also marked in (a) by the blue circle). Finally, we also calculated a box-plot representation of the identified clusters and analyzed the results via a *t* test. The ***Box Plot*** shows the phase value statistics of G1 and G2 at 1660 cm^–1^ (at the Amide I peak), demonstrating that only the G1 group presents contrast higher than the substrate value and the average noise. The workflow enables automated identification of subpopulations in a vesicle mixture, which is an ongoing research topic in biology.

This case study shows how Quasar unlocks the possibility to use AI tools in the analysis of s-SNOM data sets, which was unavailable until now in a user-friendly way.^33^

### 2.4 Case Study 4—Model Fitting

Quasar and *orange-snom* aim to address another common problem in s-SNOM data analysis, the difficulty of interpreting a nano-FTIR spectrum and drawing conclusions about the local refractive index. Due to the nature of the interaction, the results do not always resemble a simple reflectance or absorption spectrum. To solve this, simulations are needed and numerous models have been developed in the past few years to describe and model the near-field interaction between the tip and the sample, and describe the image contrast and/or spectra.^2,34–36^ One of the most successful models so far is the so-called Finite Dipole Model (FDM),^25^ which simplifies the system to a conducting prolate spheroid and a half-infinite space and treats scattering as a quasi-electrostatic problem. In addition, to understand the experimental results, one has to take into account the experiment and its parameters, such as AFM tip tapping and higher-order demodulation. Additionally, an improved successor of the model can calculate the s-SNOM signal even for layered systems.^35^

The implementation of the FDM model can be challenging for the dynamically growing user community of s-SNOM. Recently, *snompy*, a new Python package was developed at the University of Manchester with the proper implementation of the existing FDM models.^37^

Building on the *snompy* package, we developed a widget for modeling and fitting material parameters using the FDM model. We showcase the usage of the ***SNOM model*** widget. As seen on Figure 5, the widget consists of a model builder and a fit inspector part. In the fit inspector (right panel), the data are shown as blue curves, while the fitted model is shown in solid red. The view is split into amplitude (top graph) and phase (bottom graph), allowing one to simultaneously monitor the fit results for the complex data set. The user can build their layered system using different dielectric permittivity terms in the model builder (left panel). Each layer is separated by an interface placeholder that describes the thickness of the following layer. After a separator, multiple permittivity terms can be added to create the desired spectral behavior. Finally, the “*Reference*” indicator has to be placed to separate the sample terms from those of the reference.

**Figure 5:**
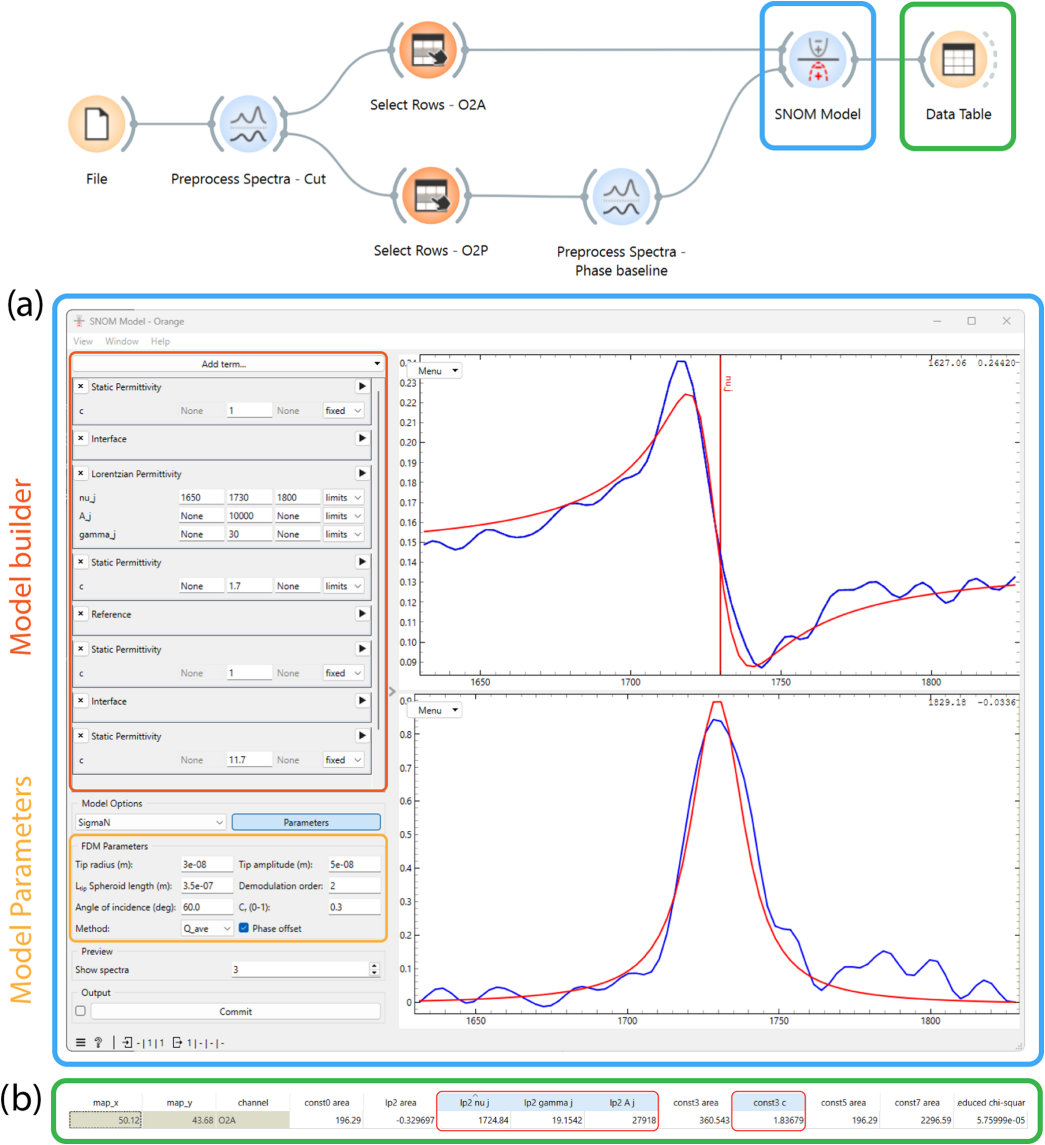
Example workflow for s-SNOM signal modeling. ***Preprocess spectra*** and ***Select Rows*** widgets were used to cut spectra and apply basic corrections on amplitude (O2A) and phase (O2P) data before model fitting. (a) Overview of the ***SNOM Model*** widget. The left side consists of a model builder part and a parameter panel. The right side shows the raw data (blue) with the fitted spectrum (red). In our demo case, we fit the complex second-harmonic s-SNOM spectrum of PET (amplitude top panel, phase bottom panel). (b) ***Data Table*** presents the fitted parameters of the Lorentzian permittivity term. The red rectangles highlight the fitted oscillator parameters as ν = 1724.48 cm^–1^, γ = 19.15 cm^–1^, *A* = ν_*p*_^2^ = 27918 cm^–2^ and a constant term which is ϵ_∞_ = 1.83.

Here, we present a model fitting of the nano-FTIR spectra of polyethylene terephthalate (PET). For the sake of clarity of the presentation, we only fit the carbonyl peak of the PET spectra. The sample was simply a piece of PET from a magnetic tape. The spectrum was measured at the SMIS beamline of the SOLEIL synchrotron. The final spectrum is the average of 16 spectra with the acquisition parameters of 80 nm tapping amplitude, 490 μm scan distance, 1024 sampling points and 20.1 ms integration time per pixel. To normalize the spectra, reference measurements were carried out on an undoped Si substrate with the same experimental parameters. After loading the data, we apply basic processing by the ***Preprocess Spectra*** widget to cut the spectrum and only keep the desired spectral region. The ***Select Rows*** helps us separate the optical amplitude (O2A) and phase (O2P) channels. We apply a basic linear baseline correction to the phase spectra to remove measurement artifacts. Amplitude and phase spectra are then separately fed to the inputs of the ***SNOM Model*** widget.

As shown in Figure 5, the simple model comprises a “*Static Permittivity*” term representing air and a “*Lorentzian Permittivity*” and an additional “*Static Permittivity*” term describing the infrared response of the PET. The extra “*Static Permittivity*” is used as ϵ_∞_ representing the response of all the other oscillators outside of the spectral range. An “*Interface*” indicator is used to separate the two domains. The initial parameters of the Lorentzian term are roughly set by hand, including upper and lower bounds of the allowed parameter set. After the reference indicator, we build a similar model consisting of a static permittivity for the air, an interface separator, and a second static permittivity for the Si with ϵ = 11.7.

The FDM model also requires several model parameters to be set, regarding the tip geometry and experimental conditions. In our example, we use standard parameters that work well with the standard platinum-coated Arrow NCPt tips from Nanoworld, which we used during our measurements. Our parameter set for the model is shown in the FDM Parameters section of the ***SNOM Model*** widget in Figure 5a. Since the negative phase offset of the experimental phase spectrum is usually removed during preprocessing, the “*Phase offset*” option can be used to offset correct the model spectrum as well.

With the parameters and the boundaries set, the widget automatically performs a fitting using both amplitude and phase data. The optimized parameters are then sent to the “*Fit Parameters*” output of the ***FDM model*** widget. They can be examined using a ***Data Table*** widget. As shown in Figure 5b the model fitting correctly identifies the oscillator properties for the carbonyl peak of PET being ν = 1724.48 cm^–1^, γ = 19.15 cm^–1^, *A* = ν_*p*_^2^ = 27918 cm^–2^. The fitted parameters align well with literature data. We note that the exact peak parameters, such as position and damping, depend on the type and degree of oxidation (aging) of PET, the average molecular weight, cross-linking, additives, etc.^38–41^ Additionally, the phase peaks in nano-FTIR tend to be blue-shifted compared to far-field FTIR absorption spectra.^42–44^

## 3 Conclusion

We have presented here a novel and unique possibility for s-SNOM data analysis by extending the *orange-spectroscopy* toolbox with new processing algorithms suited for s-SNOM data analysis and also introduced *orange-snom*, in the Orange visual programming environment, that is now packaged with other spectroscopy tools in Quasar allowing for a simple installation. We demonstrated how to solve common data processing and interpretation problems with Quasar workflows. *Orange-snom* also serves as a graphical interface to *snompy*, a Python module developed for the modeling of s-SNOM data.

We have prepared a GitHub repository to show how the underlying Python modules, *pySNOM*, and *snompy*, enable those who prefer to write line-scripts or are in need of more complex data analysis by implementing all the above examples in IPython notebooks. In the repository, we also present a standalone application built with Quasar widgets in a customized canvas, enabling the construction of specific, rigid applications in a standalone context.^22^

Finally, we emphasize that the real opportunity *orange-snom* unlocks in Quasar, which we highlighted in the multispectral case study, is that the s-SNOM specific part of any given workflow can be merely the start of a more involved data analysis scheme that can benefit from the power of machine learning methods available in Orange and other previously implemented spectroscopy tools from various Quasar add-ons.

## Data Availability

All data, data processing workflows, examples, and source codes are freely available in GitHub repositories.^22,45^ Quasar can be installed via a single installer for all major platforms available from the project’s website.^23^ Alternatively, the *orange-spectroscopy* and *orange-snom* packages discussed above can be installed as add-ons to the Orange machine learning suite manually.^46^

## Footnotes

1 Automatic data flow can be disabled in various ways, like deleting or disabling the data connection between widgets, or preventing the automatic processing of the full data set, and can be useful when working with large amounts of data.

2 In all case studies, the File widgets use the same reader called “NeaSPEC (*.nea *.txt *.gsf)” in the drop-down menu, and have to be manually set to reproduce the workflows for the current version of Quasar.
